# Book Notices

**Published:** 1879-11-20

**Authors:** 


### *BOOK NOTICES.*

THE NATIONAL DISPENSATORY, containing the Natural History, Chemistry, Pharmacy, Actions and Uses of Medicines, including those recognized in the Pharmacopœias of the United States, Great Britain and Germany, with numerous references to the French Codex, by ALFRED STILLE, M. D., L. L. D., Professor of the Theory and Practice of Medicine and of Clinical Medicine in the University of Pennsylvania; and JOHN M. MAICH, Phar. D., Professor of Materia Medica and Botany in the Philadelphia College of Pharmacy. Second edition, thoroughly revised, with numerous additions, with two hundred and thirty-nine illustrations. Philadelphia: Henry C. Lea. 1879.

Only a few months ago we briefly reviewed the first edition of this great work, which by reason of the great demand for it is already exhausted, and the second edition is now before us, enlarged and improved, being perhaps the largest work on the subject now in print, containing 1,680 large octavo pages.

The improvements and additions made to the work since the first issue are many and valuable, amounting to nearly one hundred pages of new matter. The pages are somewhat larger. The doses are given both in the troy weight and the metrical system, and the Therapeutical Index has been extended by 2,250 new references, making the total number in the present edition about 6,000.

This work brings the subject down to a very recent period, the author's preface being dated August, 1879.

WALSH'S PHYSICIAN'S COMBINED CALL BOOK AND TABLET, AND WALSH'S PHYSICIAN'S HANDY LEDGER.

The former of these is for pocket use, and admirably arranged for entries and memoranda, and contains a little walking library of information as to tables, antidotes, doses, tests, incompatibles, etc., etc.

The latter, or HANDY LEDGER, is arranged to transfer the contents of the CALL BOOK, by a short hand method, so as to give in a brief space the visits and days on which they were made, so that the accounts may be drawn and the gross amount of items shown with the least possible trouble in book-keeping. Size of Handy Ledger 300 pages. Price $3. Price of Day book $1 50. Address Ralf Walsh, 326 C street, northwest, Washington, D. C.

ATLAS OF SKIN DISEASES, by LOUIS DUHRING, M.D., professor of Skin Diseases in the Hospital of the University of Pennsylvania; physician of the Dispensary for Skin Diseases, Philadelphia Hospital, etc.; Part VI—*Syphyloderma Pustulorum*, *Erythema Nodosum*, *Seborrhœa*, *Eczema*.—Philadelphia, Lippincott & Co.

This is the sixth of a series of plates illustrative of the various phases of skin affection. We have commended in strong terms the previous numbers of the series which the publishers have sent us. We see in this issue no falling off in the admirable execution of the plates, in the neatness of the get up, and in the clear, concise and able descriptions accompanying the plates characteristic of the learned author.

Dermatological science, heretofore so backward in its development on this side the waters, may now be said to be making rapid strides and must soon take the lead of our transatlantic brethren.

A TEXT BOOK OF PHYSIOLOGY, by J. FULTON, M. D., M. R. S., England; L. P. C. P., London; Professor of Physiology and Sanitary Science in *Trinity Medical College*, Toronto; Surgeon to the Toronto General Hospital, etc., etc. Second edition, revised and enlarged, 404 pages octavo, with numerous illustrations.—Philadelphia, Lindsay & Blakiston; Toronto, Willing & Williamson.

The merit of this work consists in its plain, practical and condensed method; its freedom from redundancy and abstruse speculation, thus fitting it to the wants of the stndent as a text book during his attendance upon lectures.

THIRD REPORT OF THE BOARD OF HEALTH TO THE HONORABLE CITY COUNCIL OF THE CITY OF NASHVILLE for the two years ending December 31, 1878.—Nashville, Tenn.: Tavil, Eastman & Havill. 1879.

This is a handsome volume of 384 octavo pages. It contains a well executed map of the city and a number of valuable papers bearing upon sanitary matters, hygiene, etc.

